# A Single Center Observational Study of Spirometry Assessments in Children with Congenital Heart Disease after Surgery

**DOI:** 10.3390/medicina59040764

**Published:** 2023-04-14

**Authors:** Chien-Heng Lin, Tsai-Chun Hsiao, Chieh-Ho Chen, Jia-Wen Chen, Tzu-Yao Chuang, Jeng-Shang Chang, Syuan-Yu Hong

**Affiliations:** 1 Division of Pediatric Pulmonology, China Medical University Children’s Hospital, Taichung 404327, Taiwan; 2 Department of Biomedical Imaging and Radiological Science, College of Medicine, China Medical University, Taichung 404333, Taiwan; 3 Department of Psychiatry, China Medical University Hospital, Taichung 404327, Taiwan; 4 Division of Pediatric Cardiology, China Medical University Children’s Hospital, Taichung 404327, Taiwan; 5 Division of Pediatric Neurology, China Medical University Children’s Hospital, Taichung 404327, Taiwan; 6 Department of Medicine, School of Medicine, China Medical University, Taichung 404333, Taiwan; 7 Graduate Institute of Biomedical Sciences, China Medical University, Taichung 404333, Taiwan

**Keywords:** spirometry, congenital heart disease, surgery, children

## Abstract

*Background*: Children with congenital heart disease (CHD) have impaired pulmonary function both before and after surgery; therefore, pulmonary function assessments are important and should be performed both before and after open-heart surgery. This study aimed to compare pulmonary function between variant pediatric CHD types after open-heart surgery via spirometry. *Methods*: In this retrospective study, the data for forced vital capacity (FVC), forced expiratory volume in the first second (FEV1), and the ratio between FEV1 and FVC (FEV1/FVC) were collected from patients with CHD who underwent conventional spirometry between 2015 and 2017. *Results*: A total of 86 patients (55 males and 31 females, with a mean age of 13.24 ± 3.32 years) were enrolled in our study. The diagnosis of CHD included 27.9% with atrial septal defects, 19.8% with ventricular septal defects, 26.7% with tetralogy of Fallot, 7.0% with transposition of the great arteries, and 46.5% with other diagnoses. Abnormal lung function was identified by spirometry assessments after surgery. Spirometry was abnormal in 54.70% of patients: obstructive type in 29.06% of patients, restrictive type in 19.76% of patients, and mixed type in 5.81% of patients. More abnormal findings were found in patients who received the Fontan procedure (80.00% vs. 35.80%, *p* = 0.048). *Conclusions*: Developing novel therapies to optimize pulmonary function will be critical for improving clinical outcomes.

## 1. Introduction

Cardiac and pulmonary pathophysiologies are closely interdependent; therefore, congenital heart diseases (CHDs) are frequently associated with respiratory complications, such as abnormalities in pulmonary blood flow that result in impaired lung development [1,2,3,4]. Over 60% of children with CHD have impaired pulmonary function, especially restrictive lung patterns, both before and after surgery [1,2,3,4,5,6]. Reduced lung compliance and higher expiratory airway resistance among children with CHD compared with healthy children were also reported by Yau et al. [7]. However, a spirometry assessment before surgery is often unavailable because the patient is too young or has a hemodynamically unstable condition that is not suitable for the test. The severity and complexity of different CHDs, the different degrees of invasiveness associated with open-heart surgery, the duration of postoperative intensive care, and the frequency of chest surgeries or interventional cardiac catheterizations before and after the primary open-heart surgery might also contribute to a patient’s long-term pulmonary function. Determining associations between these factors and long-term pulmonary function is difficult. We performed this retrospective study to evaluate the spirometry assessment of different types of CHD and compared the pulmonary function of patients with acyanotic CHD and cyanotic CHD and of patients who received the Fontan procedure and other procedures.

## 2. Material and Methods

### 2.1. Study Design and Subjects

The study hospital’s institutional review board (IRB) concurred that this retrospective study was a continuous quality improvement initiative to improve patient care and did not require informed consent. This study was approved by the IRB of the study hospital (CMUH109-REC1-024).

This retrospective study was conducted from 1 January 2015 to 31 December 2017 at a regional hospital in central Taiwan. Children (older than 6 years and younger than 18 years) with CHD who underwent open-heart surgery and spirometry assessment at our hospital were enrolled in this study. The pulmonary function testing indicated for the patients enrolled in our study was part of the routine care ordered by their cardiologists (JS Chang and TY Chuang). We excluded patients with acute or chronic disorders, such as respiratory infections, asthma, chronic lung disease, genetic syndromes, and musculoskeletal problems, and those who recently underwent abdominal or thoracic surgery, which is known to affect spirometry performance. No medications affecting spirometry assessments were prescribed to our patients. The parameters measured during the spirometry assessment, including the forced vital capacity (FVC), forced expiratory volume in the first second (FEV1), and the ratio between FEV1 and FVC (FEV1/FVC), were collected using a Vitalograph Pneumotrac Type 6800 spirometer (Vitalograph Inc., Lenexa, KS, USA). Three technically acceptable forced expirations were recorded in up to eight attempts.

**Figure 1:**
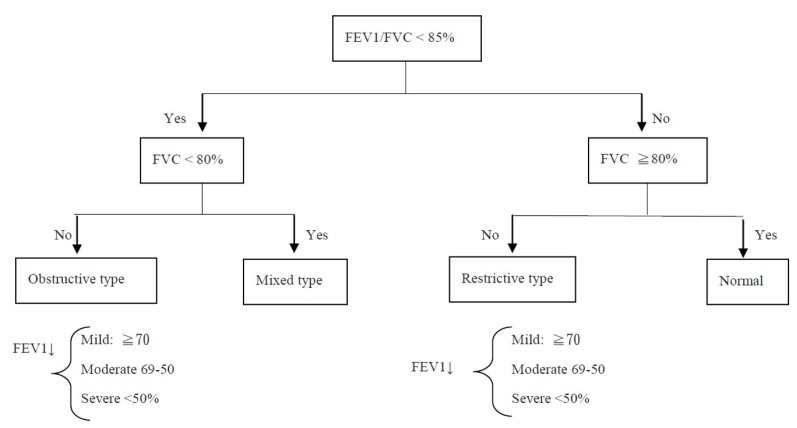
Interpretation of spirometry results. FVC: Forced vital capacity; FEV1: forced expiratory volume in the first second.

Lung functional abnormalities were classified as described in Figure 1 [8,9]. Patients with CHD were classified into five groups: atrial septal defect (ASD), ventricular septal defect (VSD), tetralogy of Fallot (TOF), transposition of the great arteries (TGA), and other diagnoses. Patients were classified into two groups: acyanotic CHD and cyanotic CHD. Cyanotic CHD involves heart defects that reduce the amount of oxygen delivered to the rest of the body, whereas acyanotic CHD does not interfere with the amount of oxygen or blood delivered to the rest of the body [10,11]. Patients were also classified into two other groups: those who received the Fontan procedure and those who received other procedures. The spirometry assessment data among these groups were analyzed and compared.

### 2.2. Analysis

Continuous variables are expressed as the mean ± standard deviation. The distinct demographic variables between the cyanotic and acyanotic cohorts or between the Fontan procedure and other procedures were verified by a chi-squared test and Student’s *t*-test. When the assumption of the chi-squared test was violated, we used Fisher’s exact test to test the categorical variable.

The collected data were not normally distributed; therefore, analyses were performed using the Wilcoxon test (paired samples). ANOVA was used to compare more than two groups. The SPSS package, version 11 (SPSS, Inc., Chicago, IL, USA), was used for analyses, and *p*-values less than 0.05 were considered significant for the rejection of the null hypothesis.

## 3. Results

A total of 86 patients with CHD who received open-heart surgery (mean age 13.24 ± 3.32 years; 55 males and 31 females) and underwent spirometry were enrolled in our study. All enrolled patients underwent spirometry >2 years after surgery (range: 2.1–12 years; median: 7.2 years). The distribution of CHD diagnoses was as follows: 19 (27.9%) had ASD, 17 (19.8%) had VSD, 23 (26.7%) had TOF, 6 (7.0%) had TGA, and 21 (46.5%) had other diagnoses. Among the group with other diagnoses, four (5.65%) had endocardial cushion defect (ECD), three (3.49%) had total anomalous pulmonary venous return (TAPVR), two (2.33%) had coarctation of the aorta (COA), two (2.33%) had double outlet right ventricle (DORV), two (2.33%) had pulmonary atresia, two (2.33%) had pulmonary stenosis, two (2.33%) had tricuspid atresia, two (2.33%) had Ebstein anomaly, one (1.16%) had partial anomalous pulmonary venous return, and one had hypoplastic right heart syndrome. The mean age at operation for the selected type of CHD in our study was 8.1 ± 6.1 years for ASD, 105 ± 15 days for VSD, 16.7 ± 8.5 months for TOF, and 21.3 ± 19.6 days for TGA. Normal spirometry was noted in 39 patients (45.3%), the obstructive type was noted in 25 patients (29.1%), the restrictive type was noted in 17 patients (19.8%), and the mixed type was noted in 5 patients (5.8%; Table 1). Eight patients had a history of lateral thoracotomy for BT shunt, and five patients had a Fontan-type procedure.

We classified 86 patients into 5 groups: ASD, VSD, TOF, TGA, and other diagnoses (Table 1). The spirometry assessment showed that the patients with ASD and TGA had obstructive-type findings, and patients with VSD, TGA, and others did not have severe findings. In other words, severe pulmonary function problems were only found in patients with ASD and TOF. The results of most identified abnormal pulmonary function tests were mild, except for patients with ASD, which were mostly moderate (Table 1).

Among our cohort, 44 patients were classified as acyanotic CHD, and 42 patients were classified with cyanotic CHD. In the acyanotic group, spirometry was normal in 11 patients, obstructive type in 10 patients, restrictive type in 11 patients, and mixed type in 4 patients. In the cyanotic group, spirometry was normal in 25 patients, obstructive type in 14 patients, and restrictive type in 1 patient, whereas mixed type was not observed.

Comparing acyanotic vs. cyanotic groups, the spirometry assessment results of the acyanotic CHD group showed more significantly abnormal findings than those of the cyanotic group (59.09% vs. 40.48%, *p* = 0.03), and more restrictive and mixed type results were observed in the acyanotic CHD group than in the cyanotic CHD group (restrictive: 30.56% vs. 2.27%, *p* < 0.001; mixed: 11.36% vs. 0%, *p* = 0.046). Most spirometry assessment abnormalities were classified as mild in the acyanotic (38.46%) and cyanotic (58.80%) groups. FEV1/FVC was significantly higher in the cyanotic group (85.71 ± 14.99) than in the acyanotic group (79.38 ± 17.71; *p* = 0.035). However, FEV1 did not differ significantly between groups (Table 2).

Comparing the Fontan vs. other procedure groups, the spirometry assessment results of the Fontan group showed more significantly abnormal findings than those of other procedure groups (80.00% vs. 35.81%, *p* = 0.005) (Table 3).

## 4. Discussion

Children with CHD generally have normal lung volumes and resistance to airflow when they are infants, but the elastic properties of the lungs become disturbed when the lungs are plethoric due to high pulmonary artery pressure and increased blood flow, resulting in lung stiffness [12]. The elastic properties of the lungs can be altered by the performance of heart surgery [13]. Our study showed that 54.65% of patients with CHD continued to present abnormal pulmonary findings even after surgery, and children with CHD after the Fontan procedure had significantly more abnormal findings than those who received other procedures. However, the obstructive type was observed more frequently than the restrictive type (29.1% vs. 19.8%) in our study, which differs from existing reports in the literature [12,14]. In addition, more patients receiving the Fontan procedure showed abnormal PFT results than patients who had other procedures.

Many factors may contribute to impaired pulmonary function after cardiopulmonary and cardiac bypass surgery, such as postoperative inflammation and hypercortisolemia [15,16,17]. Direct lung injury can be caused by ischemia, and inflammatory responses that follow reperfusion after cardiopulmonary bypass play important roles in lung injury [18,19]. Other contributions to lung dysfunction include alterations of the chest wall’s mechanics due to sternotomy, the presence of postoperative atelectasis, pulmonary edema or pleural effusion, and impaired respiratory efforts due to postoperative pain [20,21]. Phrenic nerve injury has also been reported to induce hemidiaphragmatic paresis or paralysis after the surgical correction of CHD, resulting in poor PFT [22,23,24].

Sulc et al. reported that the surgical repair of ASD did not result in substantial improvements in postoperative spirometry assessments and found only a small decrease in the overall frequency of PFT abnormalities [5,24,25]. Therefore, prominent lung dysfunction is likely caused by events unrelated to perioperative or postoperative factors. In our study, PFT was not performed before surgery in most patients who are typically too young or have severe respiratory conditions that preclude their ability to perform the test; therefore, we were unable to compare spirometry assessment results before and after surgery.

Kurniawan et al. reported that 60.7% of children with left-to-right shunt CHD showed abnormal lung function, among which restrictive lung function was the most commonly observed abnormality [2]. However, in our study, 54.6% of patients with CHD had abnormal lung function, and the obstructive lung pattern was more frequently observed than the restrictive lung pattern. These discrepant outcomes could be due to differences in the CHD distribution between studies in addition to differences in the surgical methods, the times of operation, and the complications after an operation. Our study included a relatively small sample size, indicating the need for further study. In addition, the patients in the study by Kurniawan et al. may not have received definitive defect treatments when they underwent spirometry testing [2,4,26,27,28].

Yau et al. found that children with left-to-right shunt CHD had lower lung compliance and higher expiratory airway resistance than normal children [7], and these results are similar to the findings in our study (59.09%). A review of the literature showed that patients with cyanotic CHD have more restrictive lung patterns after surgery. This phenomenon may be due to these patients having more complex cardiac lesions, such as TGA and tricuspid atresia, which require multiple or multi-staged operations, resulting in further lung volume restriction after surgery [27,28]. However, because of the small number of patients in our study, the percentages of the obstructive pattern and the restrictive pattern were almost the same (52.94% vs. 47.06%). In addition, in our study, CHD patients after the Fontan procedure had significantly more abnormal PFT than patients who received other procedures. The Fontan procedure is used in pediatric patients who possess only a single functional ventricle; those patients had more possibility to progress to heart failure, and their pulmonary function is relatively poorer after the operation than patients who received other procedures. 

Some limitations must be noted for our study. First, our study was performed as a retrospective, single-center study with a relatively small number of cases. We compared the results of pulmonary function tests between the four most common types of CHD (ASD, VSD, TOF, and TGA) and found no significant differences, possibly due to the small sample size. The *p*-values were not significant, possibly due to the low power and limitations of the small subgroup sizes in Table 1.

The spirometry assessment’s timing was at the clinical physician’s discretion, and only patients > 6 years could properly perform forced inspiration and expiration during PFT. Although the timing of spirometry assessments varied between 2.1 and 12 years after surgery in our study, these parameters of pulmonary function were adjusted by the predicted value depending on patient age and height. Second, we did not perform spirometry assessments before heart surgery because most patients underwent heart surgery as young infants and were too young to undergo PFT; therefore, we could not compare lung function before and after heart surgery. Third, some minor residual cardiac lesions, such as valve regurgitation and small septal defect, might also contribute to a patient’s long-term pulmonary function; therefore, these factors should be considered and analyzed in more detail. Fourth, not all patients underwent body plethysmography; therefore, we only collected PFT variables, such as FEV1, FVC, and FEV1/FVC, without the residual lung volume or total lung capacity. Further studies examining residual lung volume and total lung capacity should be performed in the future.

In conclusion, abnormal lung function was identified in 54.6% of children with CHD after surgery and in 40.48% of patients with cyanotic CHD. The obstructive type was more common than the restrictive type (29.1% vs. 19.8%), and abnormal PFT was more commonly identified in patients with CHD who received Fontan-type procedures than in patients who received other procedures. A prospective study that compares the results of pulmonary function tests in different types of CHD and different surgical treatments is needed. The development of novel therapies to optimize pulmonary function under abnormal circulatory conditions after cardiac surgery is crucial for improving clinical outcomes.

## Figures and Tables

**Table 1:** Demographic and clinical characteristics of the patients at baseline.

	ASD (*n* = 19)	VSD (*n* = 17)	TOF (*n* = 23)	TGA (*n* = 6)	Other (*n* = 21)	All (*n* = 86)	*p*
Age (years) (M ± SD)	15.13 ± 2.81	10.20 ± 2.42	7.85 ± 1.07	9.88 ± 4.59	10.64 ± 4.04	13.24 ± 3.32	0.15
Sex (female) (no. (%))	6 (31.58)	6 (35.30)	7 (30.46)	1 (16.67)	11 (52.4)	31 (36.05)	0.86
Type (no. (%))							
Normal	10 (45.30)	12 (70.58)	7 (30.40)	3 (50.00)	7 (33.33)	39 (45.30)	0.16
Obstructive	9 (29.06)	2 (11.76)	6 (26.08)	3 (50.00)	6 (28.57)	25 (29.06)	0.43
Restrictive	0 (0.00)	3 (17.64)	6 (26.08)	0 (0.00)	7 (33.33)	17 (19.76)	0.44
Mixed	0 (0.00)	0 (0.00)	4 (17.39)	0 (0.00)	1 (4.76)	5 (5.81)	0.68
Pulmonary Function (M ± SD)							
FEV1 (L)	2.44 ± 0.77	2.85 ± 0.82	2.49 ± 0.80	2.14 ± 0.53	2.05 ± 0.82	2.35 ± 1.01	0.56
FEV1/FVC (%)	80.10 ± 15.65	85.71 ± 12.87	78.38 ± 17.71	63.00 ± 28.01	80.29 ± 14.22	83.71 ± 12.87	0.93
Severity (no. (%))							
Mild	3 (15.79)	4 (23.52)	9 (39.13)	2 (33.33)	10 (47.62)	28 (33.72)	0.88
Moderate	4 (21.05)	1 (5.88)	4 (17.39)	1 (16.66)	4 (19.05)	14 (23.21)	0.52
Severe	2 (10.53)	0 (0.00)	3 (13.04)	0 (0.00)	0 (0.00)	5 (5.81)	0.74

ASD: Atrial septal defect; VSD: ventricular septal defect; TOF: tetralogy of Fallot; TGA: transposition of the great arteries; PFT: pulmonary function test; FVC: forced vital capacity; FEV1: forced expiratory volume in the first second.

**Table 2:** Comparison of spirometry data between the two types of CHD in our patients (acyanotic vs. cyanotic).

		Acyanotic (*n* = 44)	Cyanotic (*n* = 42)	*p* (Comparison)
Sex	Male	30 (68.18%)	29 (69.05%)	0.931
	Female	14 (31.82%)	13 (30.95%)	
Age (years)		12.01 ± 1.21	14.51 ± 0.51	0.889
Weight (kg)		45.21 ± 5.71	38.01 ± 4.22	0.752
Height (cm)		151.01 ± 2.36	149.01 ± 4.25	0.764
PFT	FEV1 (L)	2.44 ± 0.82	2.01 ± 0.81	0.271
	FEV1/FVC (%)	85.71 ± 14.99	79.38 ± 17.71	0.035
PFT	Normal	18 (40.91%)	25 (56.82%)	0.083
	Abnormal	26 (59.09%)	17 (40.48%)	0.003
Severity, *n* (%)	Mild	10 (38.46)	10 (58.80)	0.456
	Moderate	8 (30.77)	2 (11.76)	0.062
	Severe	8 (30.77)	5 (29.44)	0.073
Type	Obstructive	10 (62.5%)	9 (52.94%)	0.571
	Restrictive	1 (6.25%)	8 (47.06%)	0.042
	Mixed	5 (31.25%)	0 (0.00%)	0.021

CHD: Congenital heart disease; PFT: pulmonary function test; FVC: forced vital capacity; FEV1: forced expiratory volume in the first second.

**Table 3:** Comparison of spirometry data between patients under the Fontan procedure and those under other procedures in our patients.

		Fontan Procedure (*n* = 5)	Other Procedures (*n* = 81)	*p* (Comparison)
Sex	Male	3 (60.00%)	52 (64.19%)	0.084
	Female	2 (40.00%)	29 (35.81%)	0.056
Age (years)		12.4 ± 4.35	12.8 ± 6.21	0.752
Weight (kg)		44 ± 6.51	39.811 ± 5.21	0.452
Height (cm)		145.01 ± 3.71	155.01 ± 6.53	0.334
PFT	FEV1 (L)	2.54 ± 0.52	2.54 ± 1.32	0.452
	FEV1/FVC (%)	75.66 ± 14.99	80.48 ± 15.22	0.655
PFT	Normal	1 (20.00%)	52 (64.19%)	0.048
	Abnormal	4 (80.00%)	29 (35.81%)	0.005
Type	Obstructive	1 (20.00%)	18 (62.07%)	0.035
	Restrictive	1 (20.00%)	8 (27.58%)	0.042
	Mixed	2 (40.00%)	3 (10.35%)	0.232

PFT: Pulmonary function test; FVC: forced vital capacity; FEV1: forced expiratory volume in the first second.

## Data Availability

The data presented in this study are available on request from the corresponding author.
